# Creating musical life reviews with older people: a community case study

**DOI:** 10.3389/fpubh.2024.1249124

**Published:** 2024-01-24

**Authors:** Avi Gilboa, Nomi Levy

**Affiliations:** Music Department, Bar-Ilan University, Ramat Gan, Israel

**Keywords:** older people, musical life reviews, community, community music therapy, ripple effect

## Abstract

Older people living in their homes might experience growing loneliness, detachment from their social environment, and decreased functional ability. In this *community case study*, we report on a project we initiated to enhance the functional ability of older people by creating musical life reviews (MLR) with them. We connected seven of our music therapy graduates (MT) to older people living in the neighborhood across the street from campus. MTs were first trained to work by a protocol for creating MLRs with older people, developed by the authors of this article. They then worked with older people from the neighborhood for 10 one-on-one sessions, on personally tailored MLRs. MTs kept on meeting in weekly group supervision sessions, thus learning from each other- and forming a community of their own. Participants expressed their high satisfaction with the process and reported that their MLRs became increasingly important to them. Most of them were interested in taking their MLR one step ahead, and playing it to family and/or friends, and, as part of the process, planned a personal event to do this. Further, two big community events were initiated by participants and MTs. To conclude, we show how the community project enhanced the functional ability of those participating in it. We also point at possible challenges and recommendations for further implementation of the project.

## Introduction

It was almost by chance that we learned of the existence of the Revivim community center for older people living in the neighborhood where our university is located. When we visited there for the first time, we were surprised to discover how vibrant the center was, offering various activities for the retired people living in the neighborhood. We were also surprised to see how close this center was to our music therapy training program, a mere 10-min walk away. The potential for music-oriented collaboration was great, and after meeting with Revivim center's^1^ Activity Coordinator (AC), and members of the center, we came up with several ideas that could be implemented either at Revivim center or in our well-equipped music therapy ward. Revivim center was interested in having more music and musical activities for their members, and we were interested in connecting our music therapy students and graduates with this community of older people. In other words, both parties wanted to become good neighbors, and wanted music to lead the way in forging this relationship.

In this article we will describe one of the projects that we conducted with Revivim center, namely, the *musical life review project*. As part of her MA thesis, the second author of this article developed a protocol for creating musical life reviews with older people (1). As part of her PhD project^2^, she expanded the idea and training music therapy graduates (MTs) to use the protocol as part of a larger community-based project with Revivim center members. Both authors have had experience with community music projects and had great motivation for them to succeed. Both have a strong humanistic identity with warm feelings toward communities and strong faith in the power of music to help communities grow. Nevertheless, throughout this project we held an open approach, and enabled things to unfold in a natural way. We were ready to experience any outcome and to deal with it in the most professional way.

We were, therefore, very happy to acknowledge that the project was very successful and even after it formally ended, it had a long-lasting impact on the Revivim center community as well as the MTs that were involved in it, and on their professional community. We believe that the working model that was developed here can be implemented in similar social environments in which student communities and communities of older people want to bond using music. Before describing the project, we will give some context about the population we worked with, namely older people who live in the community, and their needs and challenges. We will also review literature referring to the technique we used in this project, namely, musical life reviews (MLRs).

## Detail to understand key programmatic elements

Many older people, even those who are generally healthy and independent in their daily functioning, experience changes, loss, separation, and a decline in different functions (2). They have reported reduced energy levels, reduced physical and cognitive abilities, a decrease in their socioeconomic status, the death of spouses, relatives and friends, and gradual separation from their children (3). These difficulties often cause older people to feel that they are less in control of their lives, and it adversely affects their self-confidence and self-esteem. Some older people suffer from symptoms of stress, anxiety, and a general difficulty finding peace of mind (4). Others experience growing social isolation, sadness and despair, and depression is reported as one of the main problems for older people (5, 6). With rising life expectancies and growing rates of healthy and independent older people in the community, it is important to seek ways to preserve their quality of life and wellbeing and to develop practices that address the growing psychological, social, and mental needs of this population (7, 8).

Music has been used in many different ways to assist older people, and to meet their psychological, physical, and mental needs (4, 9–11). For instance, singing in a choir or playing in a musical ensemble can boost vitality and energy levels, and lower depressive symptoms (6). The social nature of making music in a group can naturally promote friendships and help those who feel lonely (12). For some, an ensemble can become a small community to which they feel an affinity. Other uses of music with older people are based on the connection between music and movement. Here, music is used to encourage movement and dance, which are important for the preservation and improvement of physical abilities (13). Other uses of music are based on the benefits of enjoyment that stems from people listening to music together and especially choosing the songs they like. The act of choosing music can be very significant, because it encourages participants to share their musical taste, and sometimes the personal stories and circumstances that are connected to the chosen song (9).

Another line of techniques that has been used successfully with older people includes musical life reviews and reminiscence [see Istvandity (14) for a systematic review]. Studies show that music has the power to encourage reminiscing among older people (15–18), an ability that is essential for developing life stories. El Haj et al. (16) found that memories are retrieved more quickly and more spontaneously when music (chosen by the participant) is played, compared to retrieving them in silence. They also found that the music-oriented memories were more specific, accurate, and detailed, compared to those elicited in silence, and that they more often connected to emotional responses. Other brain research-oriented studies found connections between listening to self-chosen music and heightened abilities to reminisce, and that (mostly positive) emotional responses were also involved (17, 18).

The idea that reminiscing and life-reviewing (not connected necessarily to music) could be used beneficially in therapy was noted by Butler (19), a psycho-gerontologist. He noticed that his clients frequently and spontaneously reminisced and reviewed their lives and he realized the healing potential this had. Life reviews at this point in life can enable people to make peace with their lives, to resolve unfinished business and past conflicts, and to invest their energies in relationships they feel are important to them now. It is an opportunity to summarize and organize memories from childhood, adolescence, and adulthood, and to put together a coherent and meaningful life story (20). These ideas resonate with Erikson's (21) eighth and final stage in his psychosocial model—integrity vs. despair—which is relevant to older people in the final phase of their lives. Erikson argued that forming life reviews at this stage in life can help to enhance feelings of integrity and acceptance and to avoid feelings of regret, guilt, and bitterness that typically lead to despair. By connecting and organizing sporadic memories, one creates coherence between past, present, and future, and makes sense of life as a whole (22). Indeed, more recent studies have shown that treatments that are focused on life reviewing and reminiscing have positive effects on the psychological wellbeing of older people (23–27).

Several studies have reported using music in combination with reminiscence therapy (4, 27–31). Istvandity (14), who systematically reviewed five of these studies, noted that although reminiscence can benefit older people in general, most of the studies targeted only those with dementia. Istvandity (14) also noted that these studies did not specify the exact protocol for working with music to induce reminiscence. Therefore, other practitioners who want to implement this treatment with their clients will find it difficult to replicate. Lastly, most of these studies focused on reminiscence, not on life reviews—essentially different phenomena (29). Reminiscence is a more sporadic process where one spontaneously recalls life memories, mostly good ones, while life reviews are more systematic, with the aim of encouraging self-integration of one's life events.

In this article we will describe how we implemented MLRs with older people living in the community. As a community case study the goal of this article is to describe this community project and to explore the possibility that it enhanced general constructs such as participants' functional ability and their general wellbeing. We will first describe the protocol and how we developed it, and we will then describe how we implemented it as part of a larger community project.

## Context

### MLRs for older people—A pilot to consolidate a protocol

**Figure 1:**
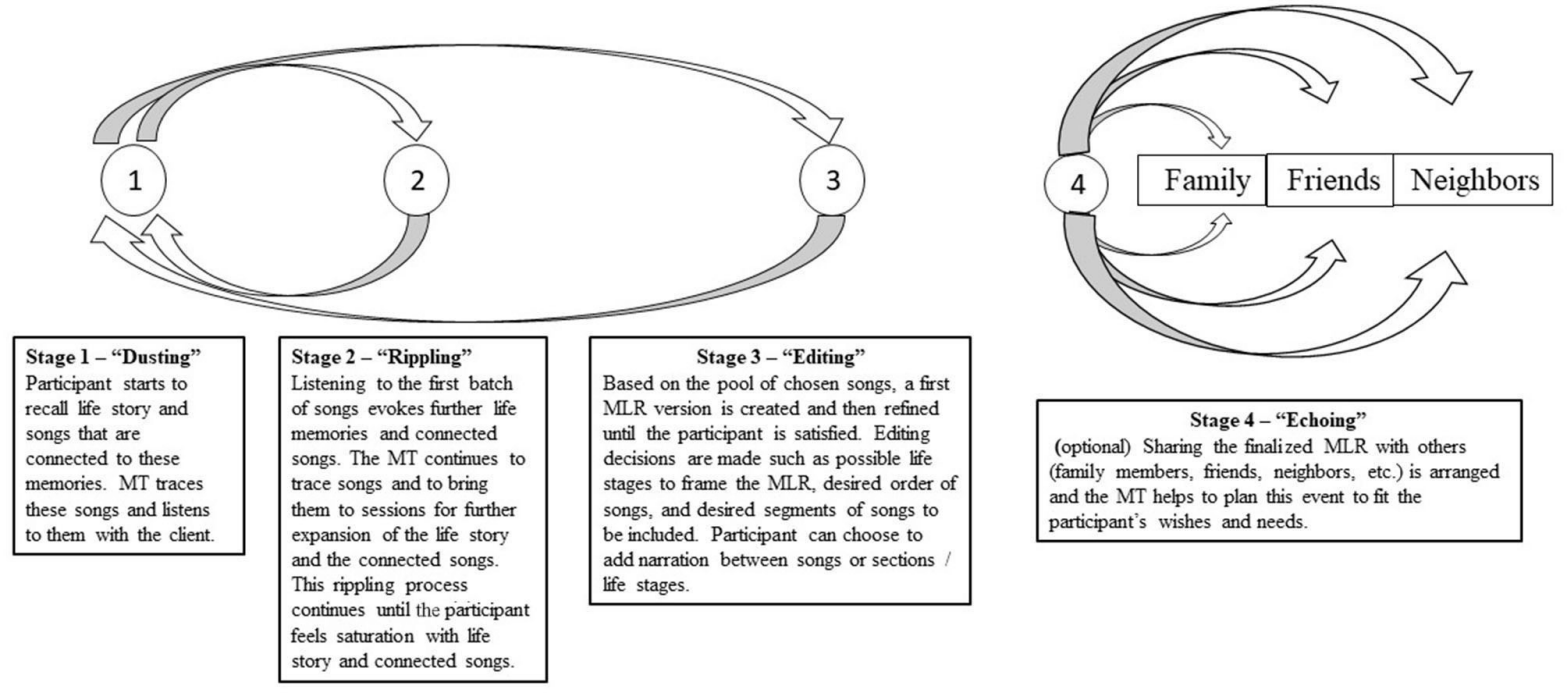
Four-stage protocol for creating MLRs with older people.

We started using MLRs with older people in a pilot project we conducted during the years 2012–2014 in a sheltered housing center. This project was documented and researched as part of the MA thesis of the second author of this article (1)^3^. The MLR protocol we consolidated for older people was based in part on the musical presentation model (MP) developed by Amir (32), used mostly in a group context with various populations (33, 34)^4^. After piloting and experimentation, we came up with a short-term protocol (8–10 sessions long; see Figure 1). Although the protocol is described in four distinct stages, the actual process required much back-and-forth maneuvering:

#### Stage 1—“Dusting” (~ sessions 1–2)

The MT encourages the participant to introduce their life story or parts of it, and to start recalling musical pieces from their past that might be connected to different parts of their life story. At this initial stage, participants remember only segments of the songs, sometimes a verse or two of the text and sometimes part of the tune that they try to hum. Some participants might have lived in different countries throughout their lives, and so with them, a variety of languages and musical styles is expected. The MT is required to work like a detective in finding leads for retrieving a recording of every requested song, even if it is rare or difficult to locate. Listening to such a retrieved song can come as a delightful surprise for participants after many years of not having the opportunity to hear it.

#### Stage 2—“Rippling” (~ sessions 3–5)

Listening to the first few songs that were found often elicits feelings of nostalgia, memories, sights, and stories that are connected to the participant's life story, and this is all documented by the MT and used to assemble more information for the MLR. Typically, one song triggers the recall of other songs and episodes. Therefore, stage 2 is actually intertwined in a back-and-forth manner with stage 1. The dynamics of these two stages remind us of ripples: One circle of memories evokes the next ripple of memories, and the process continues for several sessions when songs and stories accumulate until the participants feel they attained saturation.

#### Stage 3—“Editing” (~ sessions 6–8)

When participants are satisfied with the materials, editing begins with the aim of creating the first version of the MLR. Important decisions are made here, such as should the MLR be based on “chapters” according to stages in life, and if so—what are the participants' meaningful stages in life? What version of a song should be used, and which segment of it should be chosen? In what order should the songs be organized and grouped? Typically, a 20-min time limit is given, implying that these decisions significantly impact the form and design of the MLR. After making some tentative decisions, the MT prepares the first version of the MLR, and then plays it to the participant. The participant can then either recall more musical pieces that need to be added to the MLR (returning to stage 2 of the process) or delete or shorten songs. Listening to the first version can also trigger the retrieval of other life periods that the participant thinks need to be represented, and so, another wave of songs is revealed, and more searches are required from the MT. A second version is created, listened to for further editing, and this process repeats itself with each new version until the participant feels satisfied with the MLR. Another important decision the participant can make, typically at the end of this stage, is whether to add verbal narration between songs or life stages, providing the listener with context or telling relevant stories. When the last version is finalized, the MLR is either burned on a CD or saved as a file, to be used by the participants. A booklet can also be added with song lyrics, associated stories, etc. Typically, the participant is the one making all editing decisions and the MT is the one doing all of the technical work (usually based on software such as Shazam and Youtube to retrieve music and Audacity or Wavepad to edit the MLR). The MT also serves as an advisor in editing decisions such as how and when to fade in or out of a song, etc.

#### Stage 4—“Echoing” (~ sessions 8–10)

Although this is an optional stage, most of our participants were indeed interested in pursuing it. Typically, as stage 3 progresses, participants express their desire to share their MLR with others (e.g., family members or friends). This can be done in different ways, and the MT needs to be very attentive to the exacts needs that the client is expressing and to be creative at thinking of ways to pursue the plan. Together they decide who the audience should be (e.g., close family? friends? neighbors?); What type of event they want and how big/small they want it to be (e.g., brunch at home? Birthday celebration at one of the children's homes?); Should the MLR be distributed to the audience members and if so—in what format (CD? link to YouTube? Addition of a small booklet?).

The responses of the pilot participants were very positive and showed how deeply connected the participants were toward their MLR: “The more I hear it [the MLR] the more I love it…I don't want to change anything anymore…that's me.” For some it was a way to make peace with past stages of their lives: “our sessions caused me to raise good memories from my childhood… I could say that as an 7–8 year old—I was happy….”

### MLRs for older people—Expanding to the community

**Figure 2:**
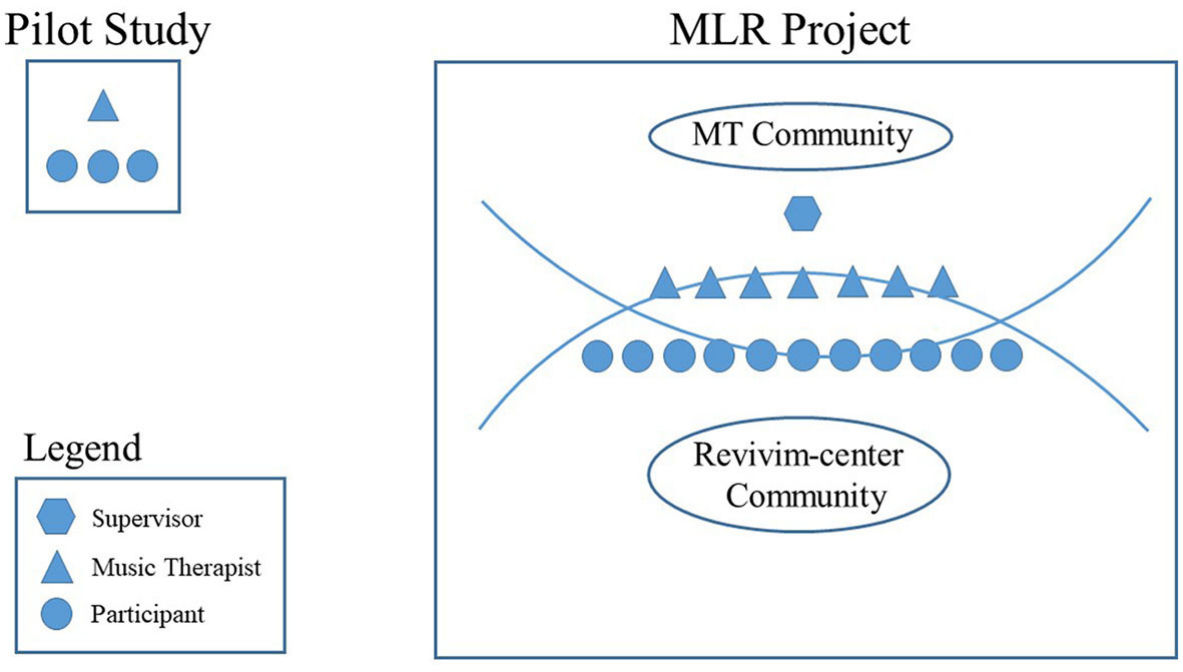
Illustration of the pilot study vs. the MLR project.

About a year after the pilot project ended, we made the connection with Revivim center and their Activity Coordinator (AC), described earlier in this article, and agreed to embark on a larger scale MLR project involving at least ten older people. Figure 2 shows that the leap between the pilot study and the community based MLR project was not only a matter of growing in numbers (from 3 participants in the pilot to 11 participants in the project). It was also a matter of adding a new group of MTs to the process, training and supervising them, and forging the bond between the MT academic community and the Revivim center community. We will describe this process stage by stage^5^.

#### Recruitment

Seven MT graduates were recruited for the project. They were given an explanation about the idea of MLRs with older people and that they would be working with Revivim center members. They would undergo a brief training period with the second author of this article, after which they would be working at the homes of older people on a one-on-one basis for about 10 sessions, to create personal MLRs. They would receive a modest scholarship for each such project based on a donation the university had for music community projects^6^.

#### Training

Training consisted of two 4-h sessions. In the first session the idea of the MLR was explained, along with information about the importance of music and life reviews for older people. MTs were asked to prepare a pilot MLR with an older family member (e.g., parent, uncle or aunt, grandparent, etc.). The next session was dedicated to listening and analyzing these MLRs, and especially the process that the MTs went through to produce it. The MTs later reported that this pilot MLR was very important for their training, for gaining confidence before they started working with “real” clients^7^.

#### Work

With the assistance of the Revivim center's AC, MTs (all Israeli) were then assigned to older people from the Revivim center who expressed their interest in creating a personal MLR. Participants were culturally diverse: they were either born in Israel or in different countries and immigrated to Israel earlier in their lives. Though MTs were familiar with various musical styles and genres, through the work on MLRs, they fine-tuned to the exact musical styles and genres that their participants knew and loved. MTs worked at the homes of these people on a weekly basis for about 10 sessions, until the MLR was finalized. Throughout these sessions, MTs continued to meet for group supervision with the second author of this article on a weekly basis. During supervision, they raised questions, challenges, thoughts, and ideas that emerged when working with their clients. Supervision was important in the development of the MTs abilities to work with the older people, and to cope with various challenges. From one session to another, the bond between the MTs got stronger as they supported each other, shared their feelings, and gave valuable advice to each other. The clients, on the other hand, were not yet aware of each other's progress. They would start bonding only later in the process. Toward the end of this stage, clients finalized their MLRs, some of them requesting to “echo” their MLRs to family members, friends, or neighbors, and the MTs were there to facilitate this. One participant invited a friend over to hear her MLR while another invited several of her childhood friends to brunch so she could play her MLR. Other participants shared their MLR with a few family members or with an apartment full of children and grandchildren. As this stage of the project came to an end, the MTs had their last supervision meeting in which they celebrated their good work and the friendships that developed between them.

#### Community bonding

Despite the fact that the MLRs were complete, and that supervision was over, there appeared to be an urge to do something more, something bigger. The Revivim center's AC contacted the authors of this article and said that participants had expressed their desire to further share their MLRs with the entire Revivim center community, and that they wanted the MTs to be a part of this event. We were happy to hear this, and so we joined forces to plan such an event. We decided on a 2-h event at Revivim center, which all members would be invited to (approx. 100 people participated). On stage, eight of the participants presented one of the songs from their MLR, sharing with the audience why they chose that particular song and what it was connected to in their life stories. MTs also took the stage when called to do so, adding their part of the story. They also prepared a few songs that they performed live. People in the audience were very much moved by the songs and the stories (some applauding enthusiastically, some in tears). These songs and stories enabled people in the audience to get to know their peers on a completely new and deeper level: “This project made me curious about my friends in the club and we got to know each other better through listening to each other's musical life stories.” The MTs, too, felt that they were part of a big and important transformation: “I suddenly understood how much meaning these sessions had for me… I find it difficult to end this process.” They felt the warm connections with their clients expanding to the larger Revivim center community: “I will continue to listen to my client's songs for the rest of my life, and his stories will be with me forever… He let me have a taste of his world, a visit to his personal hall of glory, and so—an entire world has been revealed to me. I am so grateful for this….”

A few months later, there was apparently still an urge to share the experience with others. Together with the Revivim center's AC we thought of another idea to promote the bond between the communities. We had a one-day MT academic conference coming up, and we suggested using this platform to present the MLR project. The idea was for all of the project's participants, older people who finalized their MLRs, alongside MTs that worked with them on the project, to go on stage and talk about the project and their experiences and insights they gleaned from it. This joint presentation, in front of about 70 MTs, was very successful. For the older people it was a great privilege (for some their first time ever) to present on an academic stage, and it gave them great pride and satisfaction. The MTs involved in the project also felt fulfilled to have an academic opportunity to share their experiences with their colleagues. The MTs in the audience were highly involved, applauding enthusiastically, some of them practically in tears. As the event progressed it was clear that the communities were bonding. To end the event, a song from one of the MLRs was chosen, and all of the event's participants sang it together in unison. This event formed a steady bond between the communities, and other musical projects were initiated in the following years.

## The impact of the MLR project

The MLRs had an enormous impact on the older people. Many of them expressed their appreciation of the project on different occasions, mostly at the end of the community gatherings described above. One participant said: “I'm 84 years old now and I have done many things in my life, but this project was the most important thing I've ever done!” Another participant noted how “…experiencing the creation of the musical life story was moving and cathartic.” For many participants it was a way to strengthen their connections with their children. For example, one participant said: “my daughters told me ‘after listening to your musical life review, we got to know new things about you,”' and another participant noted that “I feel that I have left something valuable to my children, grandchildren, and great grandchildren.” Some felt that the MLR connected between them and their spouses: “when playing my musical life story to my husband, he burst into tears,” and others mentioned insights they had regarding their deceased parents: “during this process I got the opportunity to mourn my father whom I lost when I was a child” or “...through this project we rediscovered our parents' music and understood the importance of our roots and this strengthened the intergenerational ties in the family.” Finally, participants mentioned how moved they were by the warm connections they made with the MTs. For some of them, the MTs were around the same age as their children or grandchildren, and this led to a warm and affectionate relationship. For many participants, being on an academic stage was in itself an accomplishment.

The MTs also felt that the project contributed to their development. As the sessions came to an end, they shared their feelings with their MT peers during supervision or wrote down their reflections. One of the MTs shared that she became acquainted with new musical styles: “I must say that I see myself listening to the MLR we created time after time… and her musical selections are simply amazing.” Some MTs got to see how impressive their clients' lives were, and they perceived them as role models: “I thanked my client for the opportunity to meet such a wonderful, powerful, positive, and creative woman. I learned a lot from her, and I especially appreciate the love she had for her family and for her late husband. You don't usually see such love and affection after 50 years of marriage. I took this as an inspiration for my own life. And when it was time to say good-bye to her—I was in tears.” Indeed, toward the final sessions, MTs expressed how sad they were that the process had come to an end: “I am very sad that these are my last two visits to her… and I think it is sad for her as well.” One of the MTs found a way around the difficulty of ending the process and came to an agreement with her client that they would continue to meet on occasion. Indeed, as we saw earlier in the account of the community events that took place after the MLRs were already finalized, that MTs and their clients had more opportunities to meet.

## Discussion

### Threads of community music therapy

Although work on the MLRs was done on a one-on-one basis, the overall MLR project took on a form typical of community music therapy (35–40). For one, participants were recruited from the community (i.e., the Revivim center community) and after their MLRs were finalized, went back to share their experiences with their community. MTs, too, were recruited from an existing community of young MTs who studied together, and throughout the project they strengthened their bonds through supervision, and then expanded the scope of their community by sharing their experiences with other MTs. Second, the impact of the MLR had “rippling” and “bonding” effects, typical of community music therapy work (39, 40). The effect of the MLR project was first local (e.g., for the individuals participating in the project) but it then “rippled” to others (e.g., participants' families, friends, and the Revivim center community at large, MT colleagues, and the professional-academic community). On an inter-community level, the MLR project “bonded” between the Revivim center community and the music therapy academic community, a bond that did not previously exist. Third, the MLR project was resource oriented (38), giving a voice to the music and to the participants' life stories. Moreover, participants had control over the materials and acted as editors of their MLR, enabling them to structure their perception of their life story and give it meaning. Finally, the MLR project was propelled by ideas of social activism and social change, which are typical of community music therapy (38). During the creation of MLRs, participants underwent different changes in their perception of themselves and of their lives, and the MTs served as agents of this change. Evidently, participants were interested in echoing these ideas of change onward to friends, family members, and other audiences. The idea that older people could and should celebrate their life stories, and that they could use musical materials to do so, affected the audiences, and the MTs and participants together served as agents for this shift in social perception. Older people in the audience who had not yet taken part in the MLR project could start fantasizing about their own MLR, and MTs who were not involved in the project but did have a connection to older clients could start imagining the possibility of seeing them in such a resourceful light.

### Practical implications and lessons to be learned for future applications

The fact that a growing number of older people live in the community and yet experience increasing feelings of loneliness, calls for a careful examination of the social resources available in the community, and for the pursuit of new ways to locate and utilize such resources. The project described in this article takes existing social resources and uses a musical framework to extract a fuller potential from them. Although Revivim center and the university campus are practically in the same neighborhood, the social connections between the two were far from utilized. The MLR project challenged both sides, the older people from Revivim center, and the MTs from the music therapy program, to interact. It gave both sides a reason to meet on a regular basis, and it clearly depicted the desired result (an MLR). Both process and product became meaningful for both the participants and the MTs. This is what drove the enthusiastic progress throughout the project, and the subsequent ripples and echoes.

Although we did not directly measure feelings of loneliness, self-confidence, or wellbeing, we could definitely see how the project improved participants' functional ability in several different ways. First, the project provided myriad opportunities for participants to learn, grow and make decisions and thus to strengthen their autonomy, dignity, integrity, freedom, and independence. Second, it encouraged the creation of new relationships (with the MTs) and the maintenance of existing relationships (with children, partners, neighbors). Lastly, it enabled the participants to contribute to society by creating the MLRs and leaving it as a legacy to generations to come. Further research can examine whether this heightened functional ability had a positive effect on well-being, self-confidence, loneliness and other psychologically measurable constructs.

Several conditions must be met to succeed in such a project. For one, there must be a clear interest for each of the communities. Such projects tend to fall apart if they are one-sided. Second, there needs to be a devoted contact person representing each of the communities who is highly enthusiastic about the joint project. Third, much creativity and flexibility are needed in shaping the project to suit the interests of both communities. Music is multifaceted, and it can be used in many ways to achieve different goals. In our context too, musical activity can be shaped and molded to suit the exact needs of the communities. In fact, during our journey with Revivim center we tried other musical formats, such as a sing-along activity led by MTs and a drumming group for the older people and their grandchildren. Each of these formats was developed and tested, and each achieved different goals. In all cases, however, a healthy bond formed between the older people an the MT community, and loneliness was pushed aside. Fourth, persistence is required, and forgiveness for trials that fail. Finally, a funding source should be available, preferably one from each of the communities, thus promising a genuine sense of partnership.

Others that seek to create community-based musical-driven bonds between older people and academic communities will need to see whether conditions are ripe to embark on such a plan. They will probably come up with a set of ideas that are suited for their specific organizational structure and use musical activities in ways tailored specifically to their goals. Whether their project is based on MLRs or on any other musical activity—the meta-purpose is to form a good and warm relationship between the communities of older people and the university students or graduates, and to activate the social resources that are already there but have not yet reached their full potential.

### Acknowledgment of conceptual or methodological constraints

One constraint of this article is that reports from participants and MTs came during and toward the end of the project, and no follow-up was conducted. Possibly, after a year or two, different perspectives on the project would emerge, including potential criticism, or additional ideas and insights that could further improve the project.

Another constraint is that the project was evaluated by the authors of this article, and although methodological precautions were taken, there are natural biases toward seeing the positive effects and overlooking possible problems. An evaluation conducted by impartial person could have benefited the process and provided further recommendations and insights for others who want to implement a similar community-based project. Further, it is recommended that in future study of this project, various outcomes and impact measures are defined such as improving wellbeing and social confidence, and lowering feelings of loneliness.

To conclude, we believe that the community project reported in this article had powerful impacts on older people as well as on the MTs that took part in it. The MLRs gave form and shape to the community potentials that were there, waiting to be realized. This project should be seen as just a first step in an evolving process, in which music in general, and MLRs in particular, are used to empower older people's functional ability, community life, and connection to other communities. Once such processes gain momentum and there are enough people are involved in such projects, it will be the time to evaluate the effectivity of the projects using evidence-based practices. We invite readers of this article who find the project to be relevant and practical in their environments, to go ahead and to promote such community music-based projects.

## Data availability statement

The raw data supporting the conclusions of this article will be made available by the authors, without undue reservation.

## Ethics statement

The research was approved by the ethics committee of the music department at Bar-Ilan University (Approval no. B.MUS.2016-5). All participants signed informed consent forms before participating in the project.

## Author contributions

AG and NL: Conceptualization, Investigation, Methodology, Writing - review & editing. NL: Project administration. AG: Supervision, Writing - original draft.

## Footnotes

^1^To keep confidentiality, the name of the center was changed.

^2^The research was approved by the ethics committee of the music department at < Anonymized> University (Approval no. B.MUS.2016-5).

^3^Methodological precautions that were taken in this study included a constant examination of various perspectives of the findings (e.g., with an additional expert, with the advisors of the study, as well as a member check with the participants in the study). These perspectives enabled the researcher to prevent self-perceived biases and to fine-tune the protocol.

^4^The general idea in the MP model is that each group participant edits a collage of musical pieces that when presented to the other group members can tell a story about him/herself. There is a 10 to 15-minute time limit, and this means that musical pieces need to be very carefully chosen, and that audio editing is required (i.e., cutting out specific parts of musical selections and pasting them in the most favorable place in the collage). In the pilot project, we made adjustments to suit the needs and abilities of older people such as working on a one-on-one basis thus enabling technical support and a specialized pace for each participant.

^5^Methodological precautions that were taken in this study included triangulation in which information was gathered from multiple sources (e.g., documentation of the supervision sessions with the MTs, interviews with the MTs), as well as the constant examination of additional perspectives on the findings (e.g., the advisor of the study, the Revivim center AC).

^6^Some MTs chose to take upon themselves more than one MLR, and their scholarship was calculated accordingly. This explains how seven MT worked with 11 older people.

^7^At this point in the article, we have transitioned from using the word “participant” to “client” because of the nature of the more personal and one-on-one work between the MT and the older individual during this phase of the project.
